# Paired Associative Stimulation of the Temporal Cortex: Effects on the Auditory Steady-State Response

**DOI:** 10.3389/fpsyt.2017.00227

**Published:** 2017-11-08

**Authors:** Sarah Engel, Robert Daniel Heinrich Markewitz, Berthold Langguth, Martin Schecklmann

**Affiliations:** ^1^Department of Psychiatry and Psychotherapy, University of Regensburg, Regensburg, Germany

**Keywords:** paired associative stimulation, auditory steady-state response, temporal cortex, tinnitus, spike-timing dependent plasticity

## Abstract

**Background:**

Paired associative stimulation (PAS) is the repeated combination of a sensory stimulus with transcranial magnetic stimulation (TMS) in close temporal association. Recently, a study demonstrated that PAS of an auditory stimulus together with TMS of the temporal cortex is capable of changing the amplitude of auditory evoked potentials (AEP).

**Objective:**

This study examined the influence of tone duration and habituation in temporal cortex PAS as elicited by 40 and 20 Hz amplitude modulated auditory steady-state responses (aSSR).

**Methods:**

Eighteen subjects participated in two experiments, including two PAS protocols each, which consisted of 200 auditory stimuli (4 kHz) paired with temporal cortex TMS with an interstimulus interval (ISI) of 45 ms between tone onset and TMS pulse, delivered at 0.1 Hz. Experiment 1 compared auditory stimuli with different lengths [PAS (23 ms) vs. PAS (400 ms)]. Experiment 2 investigated verum vs. sham PAS. aSSR for the paired tone (4 kHz) and a control tone (1 kHz) were measured pre- and post-interventional—using 40 Hz aSSR in experiment 1 and both 20 and 40 Hz aSSR in experiment 2.

**Results:**

A statistically significant, sham-controlled decrease in amplitude was observed for the 20 Hz aSSR using the 4 kHz PAS carrier frequency in experiment 2.

**Conclusion:**

Frequency-specific effects for the 20 Hz aSSR confirm the feasibility of auditory PAS and highlight the secondary auditory cortex as its target site, introducing new possible treatment protocols for patients suffering from tinnitus. The amplitude decrease can be explained by principles of spike timing-dependent plasticity and the superposition model of aSSR.

## Introduction

Transcranial magnetic stimulation (TMS) is a non-invasive stimulation technique which uses a coil placed on the scalp to apply magnetic stimulation to possible target areas of the cortex (1). A series of TMS pulses is called repetitive TMS (rTMS), which can induce changes of excitability *via* processes similar to long term potentiation (LTP) and long term depression (LTD) (2). Paired associative stimulation (PAS) is the pairing of external sensory stimuli with TMS pulses applied to the corresponding cortical region of the peripheral stimulus capable of inducing changes in neuroplasticity (3). Based on the concept of spike timing-dependent plasticity (STDP), the effects of PAS depend strongly on the order of the cortical processing of the peripheral stimulus and the TMS pulse. If cortical neurons are stimulated post-synaptically with TMS before they are excited pre-synaptically by the sensory stimulus, synaptic connectivity is reduced *via* LTD-like effects (3). If this order is reversed, LTP-like effects are expected (3). A recent pilot study revealed that the principles of PAS apply not only to the motor cortex (4) and the primary somatosensory cortex (5) but to the human secondary auditory cortex as well (6).

Tones of a specific carrier frequency can have sinusoidally modulated sound levels. These amplitude modulated tones (AM) are used to evoke auditory steady-state responses (aSSR) in the auditory cortex (7). They are recorded in the electroencephalogram as sinusoidal waves of the same frequency as the frequency of the amplitude modulation of the tone (8). So far there is no complete understanding of the mechanism underlying the aSSR. In theory, depending on the modulation frequency different parts of the auditory cortex can be activated and the generated neural responses are thought to correspond with those of transient auditory evoked potentials (AEP) (7). For example, 40-Hz AM aSSR have a modulated sound level with a period of 25 ms. Therefore, the 40-Hz AM aSSR most likely correlates with the Pa-component, a middle latency AEP with a latency of about 25 ms (9–12). There is a lot of evidence that the source of the 40 Hz aSSR is localized in the Heschl’s gyrus, which is considered to be the primary auditory cortex (13–15), which is also be presumed to be the origin of the Pa-component (16). Equivalent to the 40 Hz AM tone, a 20-Hz aSSR may reflect the P1-component, a late AEP with a latency of 50 ms generated in the secondary auditory cortex (16).

A pilot study showed that PAS of the auditory cortex is capable to induce timing- and tone-specific inhibitory effects as indicated by amplitude decreases of long-latency AEP (6). PAS (45 ms) showed greater decreases than PAS (10 ms) [PAS protocol with an interstimulus interval (ISI) of 45 ms between tone onset and TMS pulse vs. a PAS protocol with an ISI of 10 ms] (6). Schecklmann et al. assumed that the auditory evoked signal reaches the secondary auditory cortex, which has been stimulated with TMS, after about 50 ms (6). Thus, the more pronounced amplitude reduction after PAS (45 ms) was interpreted as a consequence of the shorter interval between pre- and postsynaptic excitation as compared to PAS (10 ms) (6). The effects seemed also to be frequency specific, as the amplitude decrease was more pronounced for the 4 kHz tone which had been used for the PAS intervention in contrast to a 1 kHz control tone (6). No significant effects on the AEP were observed after 0.1 or 1 Hz rTMS without acoustic stimulation that were used as control conditions (6). In this pilot study, the paired tone had a duration of 400 ms which represents a relatively long duration as PAS of the somatosensory or motor system uses electric stimuli in the range of microseconds (3). Thus, the long duration might have contributed to the inhibitory effect. One further limitation of the pilot study was the lack of a control condition that consisted of auditory stimulation in combination with sham stimulation (6). Therefore, habituation effects induced by numerous repetitions of the presented tones could not be ruled out as a potential confounder, even if the timing-specific effects (same number of presented acoustic stimuli) argued against pure habituation effects as an explanation for the observed amplitude decreases (6). Furthermore, only effects on the secondary auditory cortex were evaluated by assessing late AEP (6).

The aims of the present work were to control for effects of the duration of the paired auditory stimulus and for unspecific effects such as habituation. For this purpose, we conducted two experiments contrasting long- and short PAS tones (experiment 1) and verum (using a defined stimulation intensity) and sham stimulation (experiment 2) for the PAS stimulation. Effects were measured *via* aSSR using 40 Hz amplitude (experiment 1) and both 40 and 20 Hz amplitude modulation (experiment 2). Therefore, effects on the primary (40 Hz AM aSSR) and on the secondary (20 Hz aSSR) auditory cortex can be evaluated.

We hypothesized that PAS of the temporal cortex can induce changes in neuroplasticity. According to the model of STDP the chosen ISI of 45 ms between tone onset and the TMS pulse will lead to an increase in amplitude of the 40 Hz AM aSSR representing the primary auditory cortex and to a decrease in amplitude of the 20 Hz AM aSSR representing the secondary auditory cortex (16).

## Materials and Methods

### Subjects and Recruitment

Eighteen students from the University of Regensburg participated in the study. We recruited all subjects by word of mouth. All volunteers received a monetary compensation and had no relevant neurological or medical disorders. Seventeen subjects completed a multiple choice vocabulary test (“Mehrfach-Wortschatz-Intelligenztest”, third edition, MWT-B) (17). Participant 18 was excluded from this test as she was not a German native speaker. Hearing function was assessed by pure tone audiometry testing seven frequencies between 125 Hz and 8 kHz (Midimate 622D, Madsen Electronics, GN Otometry, Denmark). All participants had a hearing threshold below 30 dB HL for all tested frequencies. All subjects gave written informed consent after being informed about contraindications, side effects (3), and study procedure. The study was approved by the Ethics Committee of the University of Regensburg and performed in accordance with the last revision of the Declaration of Helsinki.

### General Study Procedure

**Figure 1:**
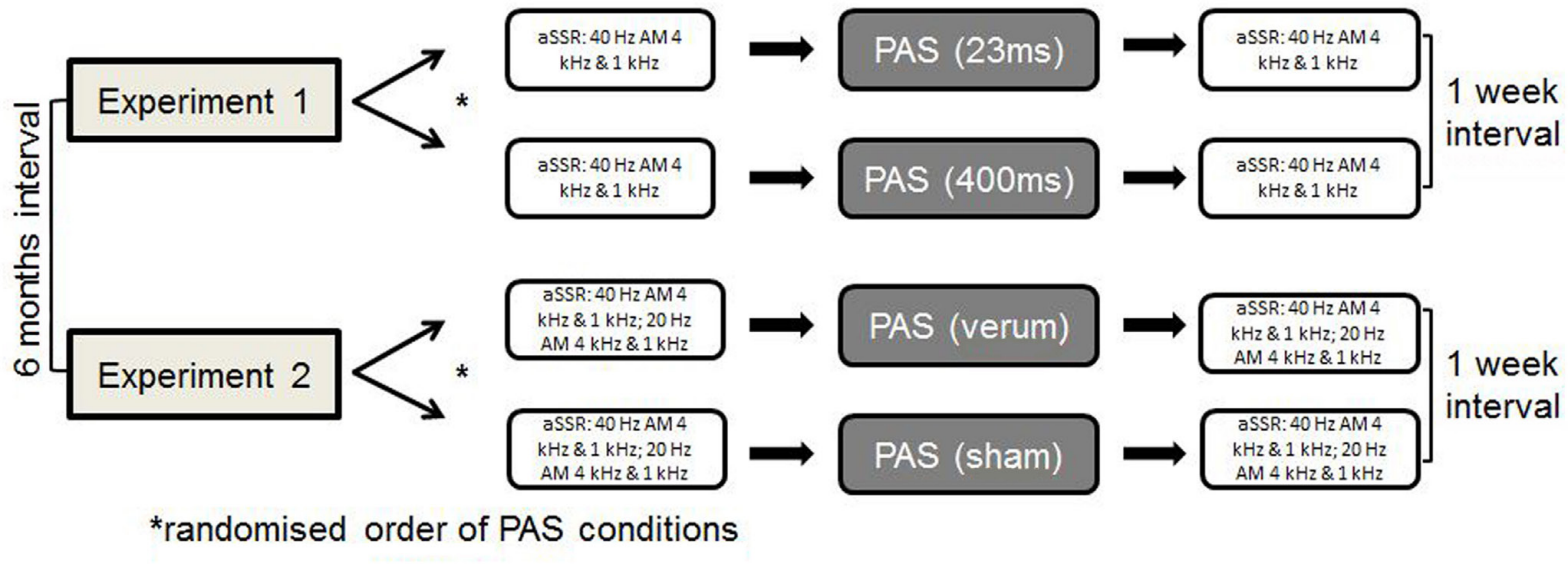
General study procedure.

All participants completed four different sessions of PAS, two within each experiments. We (Sarah Engel and Robert Daniel Heinrich Markewitz) conducted the experiments in a quiet room of the Department of Psychiatry and Psychotherapy of the University of Regensburg at the Bezirksklinikum Regensburg. One of us operated the TMS stimulator, while the other one overviewed the stimulus presenting computer program. Within experiment 1 and 2, we presented the different PAS-conditions in a randomized order 1 week apart with a 6-month interval between experiments 1 and 2. We measured aSSR before and after each PAS-condition (see Figure 1).

In the first session of each experiment, we determined the stimulation intensity [110% resting motor threshold (RMT)] for each subject following the protocol of Schecklmann and colleagues (6, 18).

For each experiment, we evaluated the sensation levels for the tones used during the experiments using Adobe audition 3.0 (Adobe Systems, DE, USA). We presented all tones binaurally through inserted earphones (E-A-RLINK, Foam Eartips for Insert Earphones, 3M, E-A-R, Etymotic Research, Inc.) at 60 dB sensation level.

### PAS Protocols

All PAS protocols lasted around 33 min and consisted of 200 stimulus pairs of an auditory stimulus of 4 kHz and a TMS pulse with an ISI of 45 ms presented with a stimulation frequency of 0.1 Hz. We used an ISI of 45 ms as the pilot study showed the largest effects for this condition (6).

**Figure 2:**
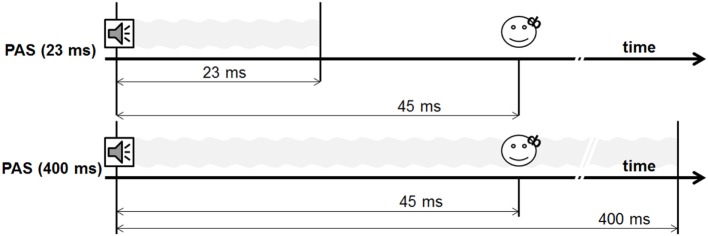
Design experiment 1.

**Figure 3:**
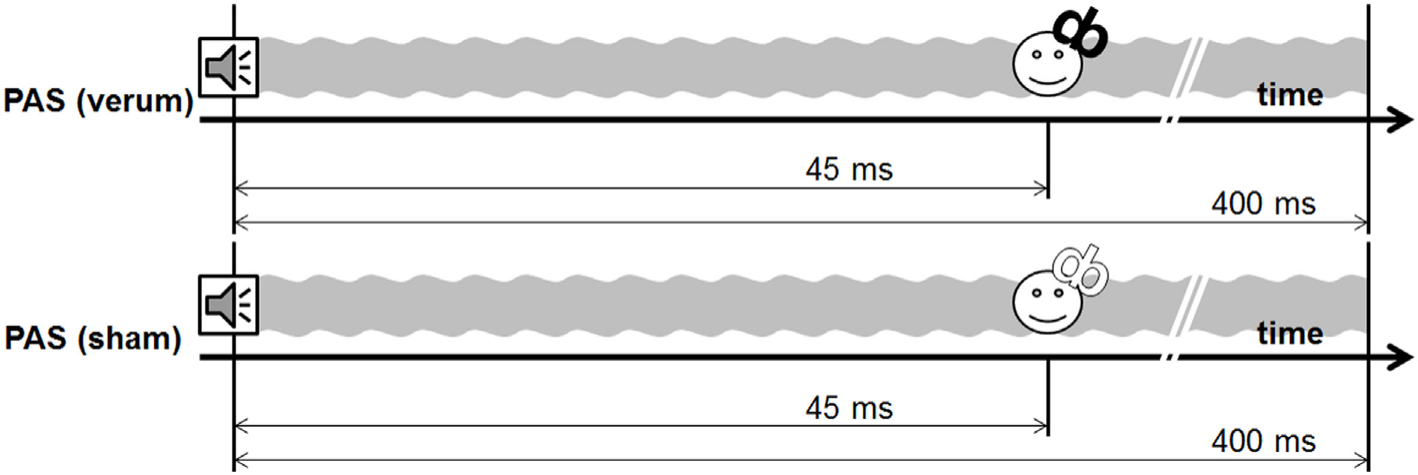
Design experiment 2.

During experiment 1, we performed two different PAS protocols, using a 400-ms tone and a 23-ms tone [PAS (23 ms) vs. PAS (400 ms)] (see Figures 2 and 3). The shortest tone length enabling a pure tone percept was 23 ms as evaluated by subjective judgment and fourier analysis as implemented in Adobe Audition.

Experiment 2 included a sham and a verum PAS protocol, using a 400-ms tone [PAS (sham) vs. PAS (verum)]. The PAS (verum) protocol was the same protocol as the PAS (400 ms) protocol used in experiment 1. In the sham condition, the water-cooled figure of eight coil was reverted in a way that the back of the coil was directed to the head of the subject. The magnetic field is decreased to one-sixth on this side of the coil as indicated by own measurements [compare technique in van Doren et al. (19)]. This sham condition guaranteed comparable sensations with respect to the click sound and the vibration of the coil.

We placed the coil over the left auditory cortex following the protocol of the pilot study (6) by using a standard procedure based on EEG coordinates (20). The TMS pulses were presented with a water-cooled figure of eight coil (MAGPRO, Medtronic, USA, outer diameter: 90 mm; water-cooled double coil). The computer software Presentation (Neurobehavioral Systems, Inc., USA) triggered the TMS pulse and presented the auditory stimuli. To ensure the exact timing of the ISI (45 ms), we measured the acoustic stimuli from the insert earplugs with a sound-level meter linked to one channel of the EEG amplifier and the TMS artifact which is induced by stimulation of the electrode cap.

### aSSR Recording and Measurement

We recorded aSSR with an EEG cap (Braincap Fast’n Easy 64 Ch for TMS, Standard Layout, Easycap, Germany), reduced impedances below 10 kΩ. We sampled EEG data with a frequency of 500 Hz (BrainAmp MR plus, Germany). We used AM tones of 800 ms duration (rise- and fall time: 75 ms) with a carrier frequency of 4 kHz (paired tone) or 1 kHz (control tone), presented in a randomized order with a variable ISI (2,800–3,200 ms). For recording EEG, we used BrainVision (Brain Products GmbH, Germany).

### Experiment 1

In experiment 1, we compared two PAS protocols, one using a 23-ms tone of 4 kHz [PAS (23 ms)] and the other one using a 400 ms tone of 4 kHz [PAS (400 ms)]. As read-out parameter we used 40 Hz AM aSSR, measured as described above, in order to evaluate the effects on the primary auditory cortex. We used two different carrier frequencies: a 4-kHz tone, correlating with the 4 kHz we used during the PAS intervention (paired tone), and a 1-kHz tone (control tone). The aSSR measurments before and after the PAS intervention lasted about 7.5 min each.

### Experiment 2

In experiment 2, we compared a verum condition (actual stimulation of the auditory cortex) with a sham condition [PAS (verum) vs. PAS (sham)]. As in experiment 1, we also used a 400-ms tone with a carrier frequency of 4 kHz for the PAS intervention. In order to evaluate the effects on the primary and secondary cortex, we measured 20 and 40 Hz AM aSSR before and after the intervention with two different carrier frequencies, 4 (paired tone) and 1 kHz (control tone). Accordingly, four acoustic stimuli were presented (40 Hz AM aSSR of 4 kHz carrier frequency, 40 Hz AM aSSR of 1 kHz carrier frequency, 20 Hz AM aSSR of a 4 kHz carrier frequency, and 20 Hz AM aSSR of 1 kHz carrier frequency). Measurements of the aSSR lasted about 15 min each.

### Data Analysis and Statistical Evaluation

We transferred all recorded EEG data to EEGLAB (21), created epochs of 4.5 s (from 2 s before till 2.5 s after tone onset), and processed the EEG data using a high (0.1 Hz) and a low (90 Hz) pass filter. After visual inspection, we excluded segments containing muscle artifacts, electrodes with signal loss, and segments with strong background noise. Further artifacts were rejected using independent component analysis.

After a subsequent visual inspection for any remaining artifacts, we interpolated the EEG data and re-referenced it to an average reference. The electrode FCz, which was used as a reference electrode during measurements, was reconstructed. EEG channels which were omitted before due to artifacts were then reconstructed using surrounding electrodes for interpolation purposes.

For the analysis of the 20-Hz aSSR, we filtered the data with 18–22 Hz, while a filter of 38–42 Hz was used for analysis of the 40-Hz aSSR.

After manually inspecting all segments of each participant for artifacts, we identified 59 as the minimum number of segments, i.e., the measurement with the smallest number of remaining segments counted 59 segments. Therefore, we used the first 59 trials of each participant and of each condition for further calculations.

Then we transferred the EEG data to FieldTrip (21). We calculated and rectified the mean voltage of all trials. Thereafter, we performed a baseline correction for the interval of 300 ms before the tone onset. We inspected the averaged and rectified trials for plausibility using topographies and trajectories. We decided to use time-locked data (averaging of the single segments) and evoked activity as the principle of STDP is related to an exact and constant timing of two stimuli.

For further statistical analysis and based on plausibility checks, we chose a time of interest of 500–800 ms to avoid interference with long-latency AEP. Our region of interest was in the fronto-parietal area (F1, Fz, F2, FC1, FCz, FC2, C1, Cz, C2). We extracted the data from these electrodes and imported it into SPSS 18.0.0 (SPSS, USA).

We computed 2 × 2 analyses of variance with two within-subjects factors “time” (pre vs. post) and “PAS-condition” (experiment 1: short vs. long tone; experiment 2: sham vs. verum condition), for both tones (1 kHz control tone and 4 kHz paired tone) and both types of AM tones in experiment 2 (20 Hz AM and 40 Hz AM). We used a two tailed paired Student’s *t*-test for *post hoc* analysis for statistically significant interaction effects. We performed corrections for multiple comparisons using Bonferroni correction.

## Results

All participants had a mean age of 21.28 years [±2.37 standard deviation (SD)] with an age range from 19 to 28 years. All participants were right handed, 10 were female. The mean hearing level (dB HL) was 13.318 ± 2.572 SD with a range (dB HL) of 8.890–17.78. There was no significant difference between the RMT for experiment 1 and 2 (*T* = −0.414; *df* = 1;17; *p* = 0.684). Experiment 1 and 2 were completed by all 18 participants without any reports of side effects.

### Explanation of Plausibility

For all subjects, we could identify the typical topography of both the 20- and the 40-Hz aSSRs as a positive maximum in the fronto-central region for the sensitive interval of 500–800 ms including the electrodes: F1, Fz, F2, FC1, FCz, FC2, C1, Cz, C2.

### Effects of PAS Adjusted for Multiple Comparisons (Bonferroni)

Experiment 1 showed no significant effects for the 1 kHz tone (all *F*-values < 5.375; all *p*-values > 0.066) and no significant effect for the 4 kHz tone (all *F*-values < 2.232; all *p*- values > 0.306).

**Figure 4:**
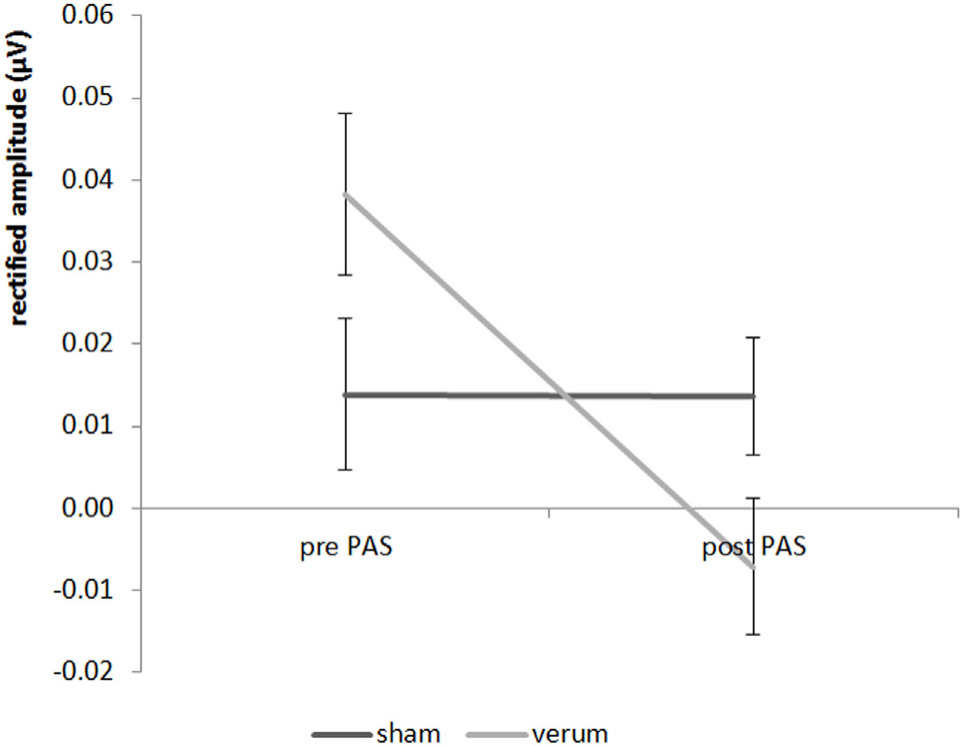
Results for the 20 Hz AM 4 kHz paired tone in experiment 2 (± standard deviation).

Looking at the results of experiment 2, we found a significant result for the 20 Hz AM 4 kHz paired tone for the main effect PAS condition (*F* = 8.816; *df* = 1;17; *p* = 0.018) as well as for the interaction “time by PAS condition” (*F* = 6.11; *df* = 1;17; *p* = 0.048) (see Figure 4), but not for the time main effect (*F* = 0.167; *df* = 1;17; *p* = 1.376). The *post hoc* paired Student’s *t*-test showed a significant decrease in the amplitude from pre to post stimulation for the verum condition (*t* = 3.505; *df* = 1;17; *p* = 0.012) but no significant decrease in amplitude of the verum condition in contrast to the sham condition after the stimulation (*t* = 2.120; *df* = 1;17; *p* = 0.196). For the sham condition there were no differences between the pre- and post-measurement (*t* = 0.253; *df* = 1;17; *p* = 1.606). Before stimulation verum and sham differed not significant (*t* = −1.374; *df* = 1;17; *p* = 0.374). Therefore, a significant decrease of the 20 Hz AM 4 kHz tone—which was paired in the PAS—could be observed for the verum condition with no changes for the sham condition. There were no significant effects for the 40 Hz AM 4 kHz tone (all *F*-values < 1.521, all *p*-values > 0.468). All effects for both 40 and 20 Hz AM tones with the carrier frequency of 1 kHz were not significant (all *F*-values < 2.188; all *p*-values > 0.314).

## Discussion

The main finding of our experiments was a significant interaction effect showing a sham-controlled PAS induced decrease of the 20 Hz aSSR amplitude. This effect was frequency specific as it occurred only for the 4 kHz tone (carrier frequency which was used for pairing in the PAS) but not for the 1 kHz control tone. We could not find any statistically significant results for the 40 Hz aSSR, neither for the 1 kHz nor for the 4 kHz carrier frequency, including experiment 1 (short tone vs. long tone) and 2 (verum vs. sham condition). The significant frequency-specific interaction effect may support the notion that PAS with combined auditory and TMS induces an inhibitory mechanism by inducing STDP. A pure habituation effect can be excluded as auditory stimulation combined with sham TMS (experiment 2) did not induce a significant amplitude reduction of the aSSR. The observed frequency specificity is in line with the results of the pilot study (6), where inhibitory effects were also observed primarily for the frequency of the tone that was paired with TMS in the PAS protocol. The frequency specificity is a further argument for the assumption that combined auditory stimulation and TMS is critical for the observed inhibitory effects.

The reduction of the 20 Hz aSSR after PAS fits well with superposition theory which explains the generation of aSSR (9–12). Based on the theory of STDP (2, 3) the PAS protocol with an ISI of 45 ms should lead to LTD-like effects for external stimuli which arrive in the stimulated cortical area later than 45 ms after auditory stimulation. Under the assumption that the 20 Hz aSSR is generated by superposition of the P1/P50 (which has a latency of 50 ms), effects of a PAS (45 ms) protocol should lead to amplitude decrease which was the case in the present study.

There were no significant effects for the 40 Hz aSSR neither in experiment 1 nor in experiment 2. As shown in the plausibility check, we were able to evoke the 40 Hz aSSR, but the PAS protocols did not induce any changes of the amplitude of the 40 Hz aSSR. 40 Hz aSSR are presumably generated by the primary auditory cortex (12), whereas the 20 Hz aSSR are most likely generated in the secondary cortex (16). Therefore, the significant decrease in amplitude of the 20 Hz aSSR as compared to no significant change in amplitude for the 40 Hz aSSR might be explained by the different anatomical origins of the 20- and the 40-Hz aSSR as described above. While the primary auditory cortex occupies most of the Heschl’s gyrus deep in the Sylvian fissure (22), the secondary auditory cortex (22), lies next to the primary auditory cortex on the external surface of the cortex. Due to its superficial location the secondary auditory cortex can be better reached with TMS than the primary auditory cortex. The individual stimulation intensity for the PAS intervention was determined as 110% of the RMT and, therefore, depended on the anatomy of the motor cortex, which lies as part of the precentral gyrus on the outer surface of the cortex as well (22). As such, we assumed that a 10% increase of the RMT will also be able to reach the auditory cortex. However, whether the stimulation intensity is high enough to have a direct effect on the primary auditory cortex is questionable. Since effects on the secondary auditory cortex could be observed, but none on the primary auditory cortex, we can assume that the intensity level of 110% of the RMT, we used during the intervention, may not be high enough to induce direct changes in neuroplasticity in the primary auditory cortex. For further experiments, we should take into account that the intensity of the electromagnetic field is inversely proportional to the distance from the TMS-coil (3).

Notably, we cannot exclude that TMS effects propagate from the secondary to the primary auditory cortex (23). However, such transsynaptically propagated effects on the primary auditory cortex would be too late to induce any STDP like effects in the investigated PAS protocols. Because we did not see any statistically significant results for the 40 Hz aSSRs neither in experiment 1 nor in experiment 2, we cannot draw any conclusions about the influence of different tone lengths of the paired tone. Further experiments using 20 Hz aSSR as read-out parameter will be needed to investigate the relevance of the tone length of the paired tone. Moreover, further experiments investigating the impact of different PAS intervals would be useful to confirm STDP as the underlying mechanism for the observed results.

Further experiments will also be necessary for additional evaluation of tonotopical effects of PAS on aSSR (e.g., using different frequencies as different stimulation conditions for the tones paired with the TMS-pulse during the intervention). Only then can an assessment of the potential of PAS as a tool both for research purposes and treatment of medical conditions be undertaken. For instance, PAS could be applied therapeutically to attenuate tinnitus symptoms. Pathogenesis of tinnitus, a phantom perception of sound (24), is thought to originate from abnormal neural activity (24, 25) and a decrease of functional inhibiting pathways (26). Our study contributes to the findings of the previous study (6) that depending on timing PAS is capable of inhibiting neural activities in the auditory cortex. Therefore, PAS of the auditory cortex could be used to reduce abnormal neural activity and to compensate the missing inhibiting pathways found in patients with tinnitus. If PAS proves to have a strictly tonotopical effect on aSSR it might even offer an individualized therapy option for people with tonal tinnitus, who could be treated with an inhibitory PAS intervention using their individual tinnitus pitch as a paired tone, other than rTMS which has shown moderate effects lasting from weeks to several months (27, 28). The potential therapeutic value of PAS in this context remains speculative.

## Ethics Statement

This study was carried out in accordance with the recommendations of the last revision of the Declaration of Helsinki with written informed consent from all subjects. All subjects gave written informed consent in accordance with the Declaration of Helsinki. The protocol was approved by the Ethics Committee of the University of Regensburg.

## Author Contributions

All authors conceived and designed the research, as well as interpreted the results of the experiments. SE and RM performed the experiments. SE drafted the manuscript and prepared figures. MS and BL edited and revised the manuscript. All authors approved the final version of the manuscript.

## Conflict of Interest Statement

The authors declare that the research was conducted in the absence of any commercial or financial relationships that could be construed as a potential conflict of interest.
