# Supplementary material for: Paired Associative Stimulation of the Temporal Cortex: Effects on the Auditory Steady-State Response
Source: Front Psychiatry. 2017 Nov 8;8:227. doi: 10.3389/fpsyt.2017.00227 (PMC5682298; doi:10.3389/fpsyt.2017.00227)
Supplement: Supplementary file 1 [file Data_Sheet_1.DOCX]

Supplementary Material

**Paired Associative Stimulation of the Temporal Cortex:**

**Effects on the Auditory Steady State Response**

**Sarah Engel*^1^, Robert Markewitz^1^, Berthold Langguth^1^, Martin Schecklmann^1^**

*** Correspondence:** Sarah Engel: sarah@engelvita.de

# Supplementary Figures
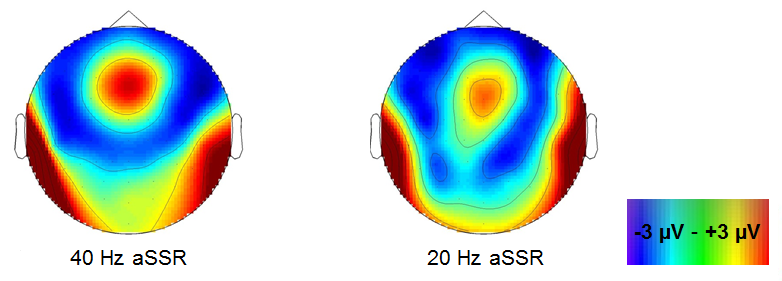
Supplementary Figure 1. Topographies for 40 Hz and 20 Hz aSSR.


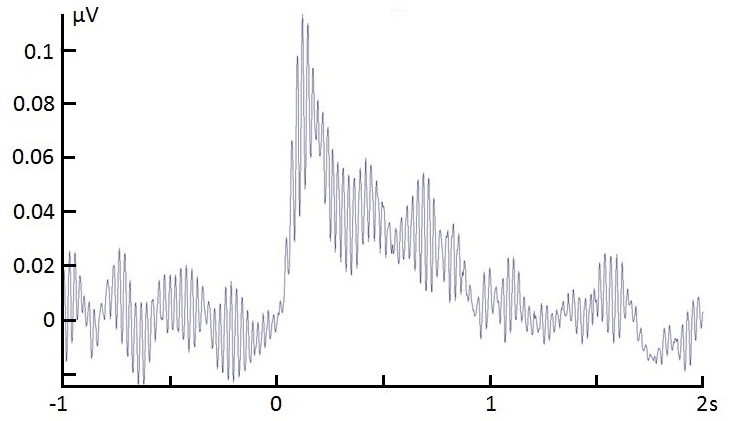


**Supplementary Figure 2.** Trajectory of the 20 Hz aSSR


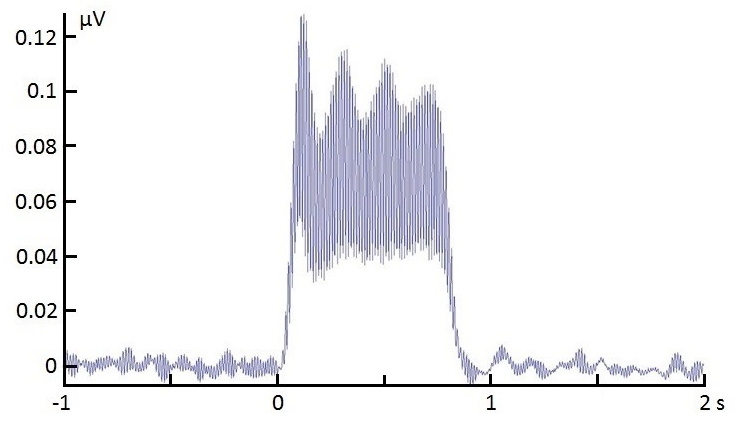


**Supplementary Figure 3.** Trajectory of the 40 Hz aSSR


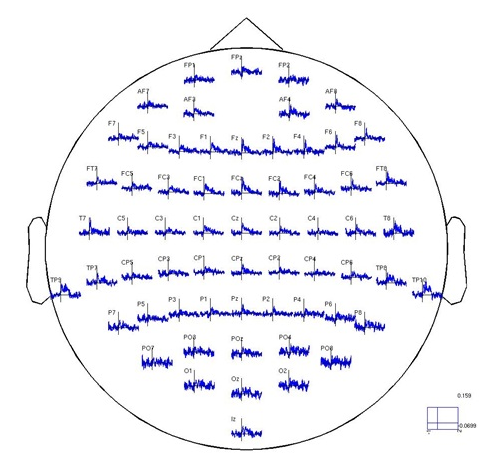


**Supplementary Figure 4.** Trajectories of the 20 Hz aSSR


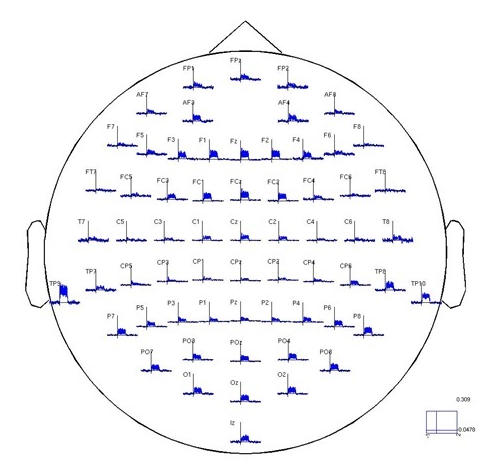


**Supplementary Figure 5**. Trajectories of the 40 Hz aSSR
